# High-intensity interval training and energy management education, compared with moderate continuous training and progressive muscle relaxation, for improving health-related quality of life in persons with multiple sclerosis: study protocol of a randomized controlled superiority trial with six months’ follow-up

**DOI:** 10.1186/s12883-021-02084-0

**Published:** 2021-02-11

**Authors:** Nadine Patt, Jan Kool, Ruth Hersche, Max Oberste, David Walzik, Niklas Joisten, Daniel Caminada, Francesca Ferrara, Roman Gonzenbach, Claudio Renato Nigg, Christian Philipp Kamm, Philipp Zimmer, Jens Bansi

**Affiliations:** 1 Department of Neurology, Kliniken-Valens, Rehabilitationsklinik-Valens, Taminaplatz 1, 7317 Valens, Switzerland; 2 Rehabilitation Research Laboratory 2rLab, Department of Business Economics, Health and Social Care, University of Applied Sciences and Arts of Southern Switzerland, Via Violino 11, 6928 Manno, Switzerland; 3 Department of Molecular and Cellular Sports Medicine, Institute of Cardiovascular Research and Sports Medicine, German Sport University Cologne, Am Sportpark Müngersdorf 6, 50933 Cologne, Germany; 4 Department of Performance and Health (Sports Medicine), Institute for Sport and Sport Science, Technical University Dortmund, Otto-Hahn-Straße 3, 44227 Dortmund, Germany; 5 Labormedizinisches Zentrum Dr Risch, Lagerstrasse 30, 9470 Buchs, Switzerland; 6 Health Science Department, Institute of Sport Science, University of Bern, Bremgartenstrasse 145, 3012 Bern, Switzerland; 7 Neurocentre, Luzerner Kantonsspital, Spitalstrasse, 6000 Luzern, Switzerland; 8 Department of Neurology, Inselspital, Bern University Hospital and University of Bern, Bern, Switzerland

**Keywords:** Multiple sclerosis, Multidisciplinary rehabilitation, Exercise, Energy management education, High-intensity interval training, Fatigue, Quality of life, Occupational therapy, Inflammation

## Abstract

**Background:**

Persons with multiple sclerosis (PwMS) often have reduced aerobic capacity and report fatigue as the most disabling symptom impacting their health-related quality of life (HRQoL). A multidisciplinary rehabilitation approach is recommended for successful management of symptoms, although there is little supporting evidence. The aim of this study is to evaluate the effect of a multimodal therapy approach, including endurance training and patient education, during a three-week inpatient rehabilitation stay, on HRQoL in PwMS at six months follow-up. Inpatient energy management education (IEME) + high-intensity interval training (HIIT) will be compared with progressive muscle relaxation (PMR) + moderate continuous training (MCT).

**Methods:**

This study has a two-armed single-blind randomized controlled superiority trial design. One hundred six PwMS-related fatigue (relapsing-remitting or chronic progressive phenotypes; Expanded Disability Status Scale (EDSS) ≤ 6.5) will be recruited at the Valens clinic, Switzerland, and randomized into either an experimental (EG) or a control group (CG). EG: participants will perform IEME twice and HIIT three times per week during the three-week rehabilitation stay. IEME is a group-based intervention, lasting for 6.5 h over three weeks. HIIT contains of five 1.5-min high-intensive exercise bouts on a cycle ergometer at 95–100% of peak heart rate (HR_peak_), followed by active breaks of unloaded pedalling for 2 min to achieve 60% of HR_peak_. CG: participants will perform PMR twice and MCT three times per week during the three-week rehabilitation stay, representing local usual care. PMR consists of six 1-h relaxation group sessions. MCT consists of 24-min continuous cycling at 65% of HR_peak_. The primary outcome is HRQoL (Physical and Mental Component Summaries of the Medical Outcome Study 36-item Short Form Health Survey; SF-36), measured at entry to the clinic (baseline, T_0_), three weeks after T_0_ (T_1_) and at four (T_2_) and six (T_3_) months after T_0_. Secondary outcomes comprise cardiorespiratory fitness, inflammatory markers (measured at T_0_ and T_1_), fatigue, mood, self-efficacy, occupational performance, physical activity (measured at T_0_, T_1_, T_2_ and T_3_) and behaviour changes in energy management (measured at T_2_ and T_3_).

**Discussion:**

This study will provide detailed information on a multimodal therapy approach to further improve rehabilitation for PwMS.

**Trial registration:**

This trial was prospectively registered at ClinicalTrials.gov (NCT04356248; 22 April 2020).

**Supplementary Information:**

The online version contains supplementary material available at 10.1186/s12883-021-02084-0.

## Background

In persons with multiple sclerosis (PwMS), reduced aerobic capacity and impaired mobility are among the most frequent symptoms [1]. In contrast, fatigue is not easily observable, but is one of the most common [2, 3] and troubling symptoms [4], resulting in impaired health-related quality of life (HRQoL) [5] in 65% of PwMS. Living with multiple sclerosis (MS)-related fatigue affects all aspects of everyday life. PwMS increasingly delegate challenging tasks at work or in the family, and fatigue is often a major reason for them to leave or change employment, which may lead to social and financial burden [6]. On the physical and cognitive level, this vicious circle often leads to an increasingly passive lifestyle and an additional loss of physical and cognitive performance among PwMS. At the emotional level, feelings of inability and dissatisfaction and the risk of greater social withdrawal and depressive mood increase [6, 7]. The causes and consequences of MS-related fatigue are thought to be multidimensional. Guidelines and systematic reviews recommend a multidisciplinary approach, which involves exercise therapy, self-management, and education, for successful management of symptoms [8–11]. However, to date, strong evidence is available only for single unimodal interventions, such as physical therapy, training or energy management/conservation programs [12, 13].

Two systematic reviews report that educational programs and approaches based on cognitive behavioural principles reduce patient-reported fatigue [12, 14], but the relevance for patients’ functioning and HRQoL remain unclear. Recently, a three-week inpatient energy management education (IEME) program for inpatient rehabilitation was developed [15] that integrates the principles of patient education, the trans-theoretical model of behaviour change, social cognitive theory, energy conservation strategies and cognitive behavioural techniques. In a randomized controlled feasibility trial [16] IEME was compared with progressive muscle relaxation (PMR) that, in previous research, improved HRQoL in PwMS [17]. The results show that IEME has a significant effect on HRQoL and self-efficacy in performing energy conservation strategies compared with PMR at discharge. This benefit slightly increased up to four months follow-up. However, it remains unclear: (i) whether this benefit is maintained up to six months; (ii) which energy conservation strategies can be applied more frequently and more easily into the patients’ daily routine; and (iii) which barriers and facilitators IEME participants have experienced during behaviour change in energy management implementation.

Exercise programs for PwMS have been described as promising supportive therapy options for reducing symptoms and side-effects of the disease [18, 19]. However, PwMS are often inactive [20]. High-intensity interval training (HIIT) is a promising, time-saving training program for PwMS that efficiently improves aerobic capacity [21–23] and fatigue [21]. Recent intervention studies show that, in contrast to moderate continuous training (MCT), HIIT reduces indicators of inflammatory activity (serum levels of matrix metalloproteinase-2 (MMP-2) [22]) and the neutrophil-to-lymphocyte ratio (NLR) [24], associated with disability status, symptomatology and disease activity. In addition, we and others have shown that physical exercise seems to influence further biological processes, such as the kynurenine (KYN) pathway, which play a crucial role in the pathogenesis and progress of MS [22].

IEME and HIIT are both proven to be more effective than the respective standard therapies (PMR and MCT, respectively), in terms of improving HRQoL, increasing cardiorespiratory fitness and reducing indicators of inflammatory activity. The combination of HIIT and IEME, which use different therapeutic approaches (training and patient education), is recommended, but has not yet been evaluated. Therefore, this study will evaluate the effects of the combination of two different three-week inpatient therapy approaches; IEME + HIIT (experimental group; EG) versus PMR + MCT (control group; CG, representing usual care at the Valens clinic, Switzerland) on HRQoL after three weeks’ rehabilitation and after returning home (at four and six months’ follow-up) in PwMS.

We hypothesize that IEME + HIIT (EG) compared with PMR + MCT (CG) will improve HRQoL at six months’ follow-up. Secondary objectives are: (i) to evaluate the effects on fatigue and mood; (ii) to evaluate specific effects of IEME/PMR on self-efficacy in using energy conservation strategies and occupational performance; (iii) to evaluate specific effects of HIIT/MCT on cardiorespiratory fitness and disease-related biomarkers (MMP-2, KYN and NLR); and (iv) to gain additional knowledge about more frequent application of energy conservation strategies and about barriers and facilitators experienced after returning home.

## Methods

### Design

**Fig. 1:**
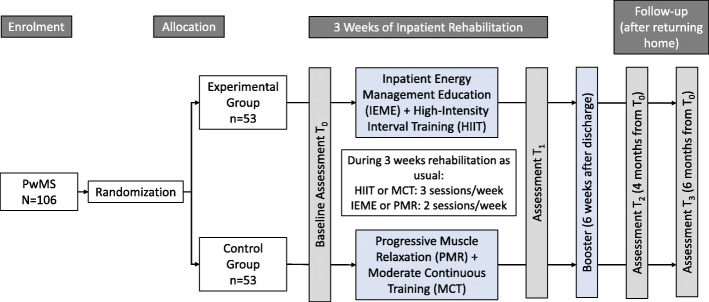
Participant flow

This study is a two-armed single-blinded randomized controlled superiority trial in PwMS referred for three weeks’ inpatient rehabilitation to the Valens clinic, Switzerland. The participants’ flow is shown in Fig. 1. Outcome measures are collected at baseline (T_0_) and three weeks later, after completing rehabilitation (T_1_). Follow-up assessments are at four (T_2_) and six (T_3_) months after baseline. Before study inclusion, participants are asked to sign written informed consent.

Participants in the EG perform IEME on two days and HIIT on three days per week. Participants in the CG perform PMR on two and MCT on three days per week. Before the three-week intervention period, a cardiopulmonary exercise test (CPET) is performed to guide the heart rate monitored training.

CPET and blood sampling at rest are conducted at T_0_ and T_1_ to evaluate the effects of the endurance exercise modalities on fitness parameters and on specific markers in the blood suspected to be influenced by training. CPET and blood sampling are conducted 24 h before the first training session at T_0_ and 24 h after the last training session at T_1_. Patient-reported outcome measures (PROMs) are completed to evaluate short-term (T_0_ vs. T_1_) and long-term between-group effects (T_0_ versus T_2_ and T_3_) of the multimodal intervention on the HRQoL and other parameters that are relevant for a satisfying everyday life (fatigue, mood, self-efficacy in performing energy conservation strategies, self-perceived competence in activities of daily living and physical activity). After discharge, all participants receive the PROMs by post for follow-up evaluation at T_2_ and T_3_. After completion, participants return the PROMs to the research office of the Valens clinic, Switzerland. All assessments performed at T_0_ and T_1_ are conducted at the Valens clinic, Switzerland. In the EG, barriers, facilitators and maintenance of implementation of energy management strategies are evaluated using online surveys at T_2_ and T_3_.

CPET and blood sampling are performed by independent and blinded assessors. Due to the nature of this study, blinding of participants towards the allocated intervention groups is very difficult.

This study was approved by the regional ethics committee (EKOS) (EKOS20/050; Project ID: 2020–00797).

### Participants and recruitment

A total of 106 persons are consecutively recruited on the day of admission by a trained exercise scientist. The recruitment period will last 15 months. Key inclusion criteria are: age > 18 years, a definitive diagnosis of MS (revised McDonald criteria [25, 26]) with relapsing-remitting, primary or secondary progressive phenotypes, an Expanded Disability Status Scale (EDSS [26]) score ≤ 6.5, a total score on the Fatigue Scale of Motor and Cognitive function (FSMC) ≥ 43 [27], and literacy and understanding in German. Exclusion criteria are: cognitive impairment (22-point Mini-Mental State Examination score (MMSE) < 21 [28]), depression subscale of Hospital Anxiety and Depression scale (HADS) > 11 [29], women planning to become pregnant/pregnant/breastfeeding, stem cell treatment in the last six months, and participation in a previous IEME or HIIT study. All included participants undergo a brief, general medical screening for study eligibility and are excluded if they have persistent infections, cardiovascular and pulmonary diseases, or if they are unable to follow the study procedures. During the study, participants are excluded if they have acute, severe relapses or withdraw from the written consent.

### Randomization

Participants are randomly allocated (1:1) to either the EG (IEME + HIIT, *n* = 53) or the CG (PMR + MCT, *n* = 53). Concealed randomization is conducted by independent employees using version 1.5.2 of the Randomization in Treatment Arms software (RITA, EVIDAT, Kiel, Germany). Randomization follows the minimization procedures according to Pocock and Simon (for review see [30]). The following stratification factors are used: age, sex, MS-phenotype (relapsing-remitting, primary or secondary progressive), disease severity (EDSS score), total fatigue score of the FSMC, levels of cardiorespiratory fitness (peak wattage (Watt_peak_)) and HRQoL (EuroQol-visual analogue scales score (EQ-VAS) [31]) at baseline.

### Study intervention

Treatment in both groups consists of education interventions combined with specific endurance exercise modalities. The highly standardized study interventions are implemented into the participants’ inpatient rehabilitation program, which is tailored to the patient’s additional needs and includes physiotherapy (flexibility, mobility, strength), occupational therapy (ability and adaptation training) and, occasionally, speech therapy, neuropsychological training or counselling by a physician or social worker.

Study interventions differ between groups regarding the applied education approaches and training intensities. Participants in both groups receive an education approach (IEME or PMR) on two days per week and exercise on three days per week on a cycle ergometer (HIIT or MCT) over a period of three weeks (inpatient rehabilitation). The education interventions in both groups (IEME or PMR) are supervised and conducted by MS-experienced and trained occupational therapists (IEME) and physiotherapists (PMR). Exercise intensities are regulated based on peak heart rate (HR_peak_), which is determined in the initial CPET at T_0_. Exercise sessions in both groups include a three-minute warm-up and cool-down at low intensity (50% HR_peak_). The heart-rate-monitored training in both groups is supervised by MS-experienced exercise scientists.

#### Experimental intervention (IEME + HIIT)

##### Energy management education (IEME)

IEME involves face-to-face educational group sessions twice a week for a total duration of 6.5 h over a three-week period. The sessions begin with an individual lecture (1 h), in which participants analyse their energy account, followed by five self-contained IEME group sessions (5 h). Sessions cover the following topics: break management, occupational balance, use of body and environment, simplifying activities and effective communication. Between sessions, participants complete assignments regarding the use of energy conservation strategies and plan the implementation of behavioural changes in their daily routine using self-training tasks. Before discharge from the clinic, the sessions conclude with an individual lecture (0.5 h), in which participants set goals concerning implementation of the learned strategies at home. The IEME-program integrates the principles of patient education, the trans-theoretical model of behaviour change, social cognitive theory, energy conservation strategies and cognitive behavioural techniques, as previously described by Hersche et al. [15]. All sessions are conducted by independent and trained occupational therapists.

##### High-intensity interval training (HIIT)

HIIT consists of physiologically-defined heart-rate-controlled cycling on a cycle ergometer, at 80–100 revolutions per minute (rpm) at 95–100% of HR_peak_ (assessed during the initial CPET). Participants perform five 1.5-min high-intensive exercise bouts at 95–100% of their HR_peak_, followed by active breaks of unloaded pedalling over 2 min, with the aim of achieving 60% of HR_peak_. After completion of the three-week training period, participants will receive an individual training plan to continue the training independently after returning home.

#### Control group (PMR + MCT)

Treatments in the control group represent usual care at the Valens clinic, Switzerland.

##### Progressive muscle relaxation (PMR)

In contrast to IEME, PMR uses enhanced mental relaxation strategies for reducing muscle tension. It consists of a series of standardized relaxation exercises in the supine position. Exercises involve 11 large muscle groups and are combined with deep breathing. Participants attend six group sessions (6 h in total) instructed by trained physiotherapists. Sessions are held twice a week over the three-week intervention period. At discharge participants are encouraged to continue performing the PMR training at home.

##### Moderate continuous training (MCT)

In contrast to HIIT, participants in the MCT perform physiologically defined heart-rate-controlled cycling on a cycle ergometer, at 60–70 rpm at 65% of HR_peak_ (assessed during the initial CPET). Participants execute continuous cycling for 24 min. After completion of the three-week training period, participants receive an individual training plan to continue the training independently after returning home.

#### Booster

Six weeks after discharge from the clinic, all participants receive a reinforcement letter that includes specific information material (a “booster”) to remind them of their individual plans for implementing the energy management strategies/PMR exercises and to reinforce implementation of the exercise training program at home.

### Outcome and assessments

After screening for eligibility and signing the informed consent, anthropometric (height, weight) and demographic data (age, sex, education, employment status, housing situation, smoking status, current medication) are collected, and three other assessments used for stratification are completed after inclusion (enrolment). After randomization to the intervention groups (EG or CG), there are four measurement time-points. A detailed schedule of the measurement time-points and assessments performed is provided in Table 1.

**Table 1:**
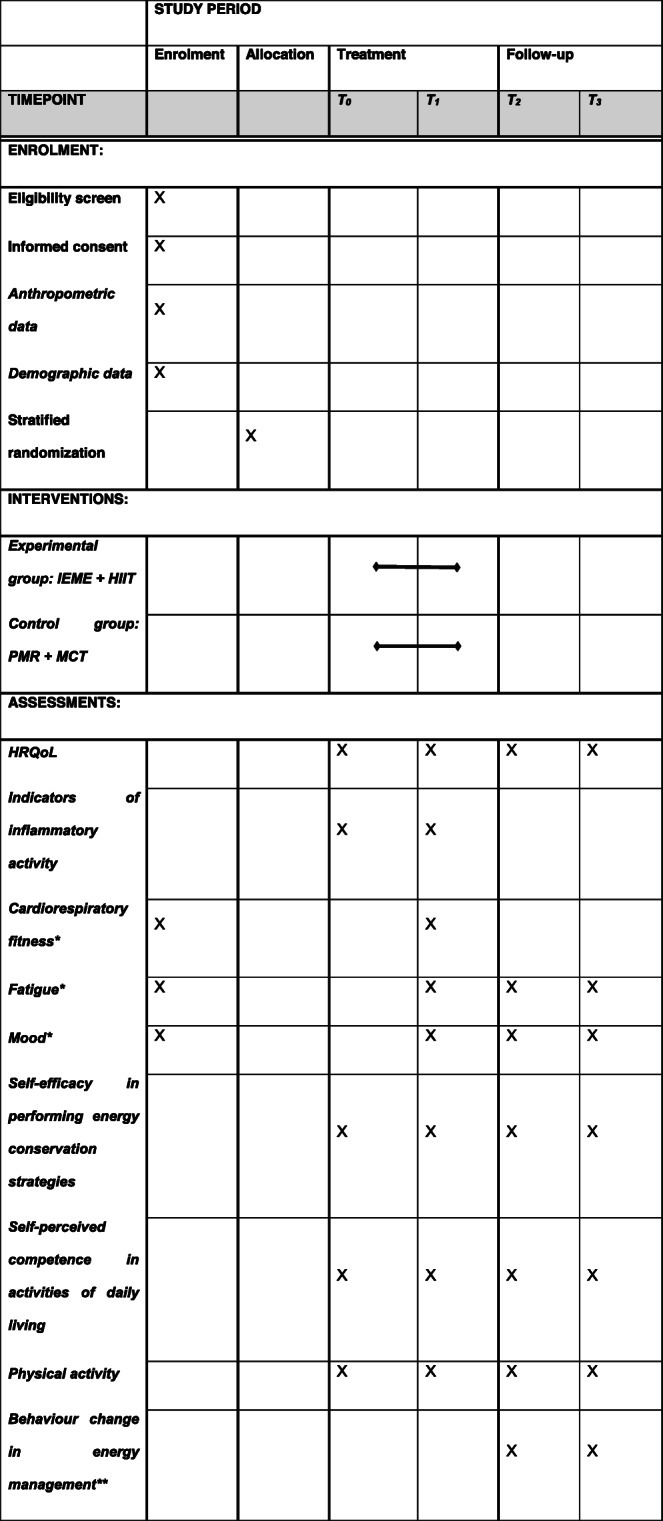
Enrolment, interventions and assessments. T_0_: baseline (entry to the clinic); T_1_: three weeks after baseline (end of intervention and inpatient rehabilitation stay, discharge from clinic); T_2_: four months after baseline; T_3_: six months after baseline; IEME: inpatient energy management education; HIIT: high-intensity interval training; PMR: progressive muscle relaxation; MCT: moderate continuous training; HRQoL: health-related quality of life.

*Cardiorespiratory fitness and fatigue are assessed before randomization, because they are used as stratification factors. Mood is also assessed before randomization because a score of > 11 in the depression subscale of the Hospital Anxiety and Depression scale would mean an exclusion from the study.

**Assessed only for experimental group

#### Primary outcome

The primary outcome of this study is the change in HRQoL over six months (T_0_ to T_3_). HRQoL is assessed using the Medical Outcome Study 36-item Short Form Health Survey (SF-36) [32]. This PROM consists of 36 items, divided into eight subscales, summarized in a Physical Component Scale (PCS) and a Mental Component Scale (MCS), with higher scores indicating better HRQoL [33]. HRQoL is assessed at T_0_ and repeated three weeks after baseline (T_1_), four months after baseline (T_2_) and six months after baseline (T_3_).

#### Secondary outcomes

Secondary outcomes evaluate the benefits of the education interventions and endurance exercise modalities.

##### Indicators of inflammatory activity (matrix metalloproteinase-2 (MMP-2), tryptophan (TRP), kynurenine (KYN), kynurenic acid (KA), quinolinic acid (QA), neutrophil-to-lymphocyte ratio (NLR), interferon-gamma (IFN-γ) and interleukin-6 (IL-6)

Blood samples are drawn at rest by vein puncture from the antecubital vein in a seated position at T_0_ and T_1_ before CPET between 08:00 and 09:00 h. To allow the participant to recover from any inconvenience caused by vein puncture, there is 15 min between sampling and the start of the CPET. Blood cell counts will be assessed using an automated haematology analyser (SYSMEX XN-1000, Norderstedt, Germany), to further determine NLR. Serum samples are centrifuged at 3000 g for 10 min at 4 °C, and the supernatant is stored at − 80 °C until analysis. Serum levels of MMP-2, IFN-gamma and IL-6 are determined using enzyme-linked immunosorbent assay (ELISA) (R&D Systems, Inc., Minneapolis, MN, USA) according to the manufacturer’s instructions. Serum levels of KYN pathway metabolites (TRP, KYN, KA, QA) are analysed by high-performance liquid chromatography coupled with tandem mass spectrometry, as described previously [34].

##### Cardiorespiratory fitness (peak oxygen consumption, peak heart rate, peak wattage)

Cardiorespiratory fitness is assessed through a CPET performed at T_0_ (before randomization, because Watt_peak_ is used as stratification factor) on a cycle ergometer (ergoline 800, ergoline GmbH, Bitz, Germany) and is repeated at T_1_. Individual cardiorespiratory fitness level is monitored by direct and continuous measurements (breath by breath) of peak oxygen consumption (VO_2peak_) by ergospirometry (Jaeger CPX, Germany). VO_2peak_ is defined as the highest VO_2_ value when the following criteria are attained: respiratory equivalent ratio (RER) > 1.10, HR_peak_ within 10 beats min^− 1^ of age-predicted maximum and rating of perceived exertion (RPE) > 8.5, as reported by Wassermann et al. [35].

##### Fatigue

Fatigue is assessed with the German version of the patient-reported FSMC scale [27], which has a proven test-retest reliability. The FSMC has defined cut-off scores to classify mildly, moderately and severely fatigued patients. Cut-off for fatigue is set at total score of 43 and motoric and cognitive sub-scores of 22. Fatigue will be assessed at T_0_ (before randomization because the score will be used as stratification factor) and will be repeated at T_1_, T_2_ and T_3_.

##### Mood

Depression and anxiety are assessed with the Hospital Anxiety and Depression Scale (HADS) [36]. The HADS is a PROM for the assessment of general mental health in adults with physical disorders, consisting of 14 items, seven for anxiety and seven for depression, with higher scores indicating anxiety or depression. HADS is performed at T_0_ (before randomization) and repeated at T_1_, T_2_ and T_3_.

##### Self-efficacy in performing energy conservation strategies

Self-efficacy is assessed with the Self-Efficacy of Perceived Energy Conservation Strategies Assessment (SEPECSA) [37]. This PROM consists of 14 items. Participants are asked to rank how confident they are that they can perform each item, on a scale from 1 (= not at all confident/sure) to 10 (= completely confident/sure). The final score is found by averaging the items, with higher scores indicating greater confidence in self-efficacy. SEPECSA is performed at T_0_ and repeated at T_1_, T_2_ and T_3_.

##### Self-perceived competence in activities of daily living

Self-perceived competence is assessed with the Occupational Self-Assessment (OSA) [38]. This PROM consists of 21 items that represent participation in habits and roles, performance of skills, and volition for participation [39]. Participants rate each item with two 4-point scales to indicate their self-perception of occupational competence (I have a lot of problems doing this – I have some difficulty doing this – I do this well – I do this extremely well) and value for importance (This is not so important to me – This is important to me – This is more important to me – This is most important to me). Following these two steps, clients review their ratings and choose areas of occupational performance and participation that they would like to change. All three steps of the OSA are performed at T_0_. At T_1_, T_2_ and T_3_ the first two steps (self-perception of occupational competence and importance) are repeated.

##### Physical activity

Physical activity level is assessed with a customized and adapted version of the Godin Leisure-Time Exercise Questionnaire (GLTEQ) [40, 41]. Additionally, two customized questions were added: (i) subjective rating of activity level over the last seven days, (ii) scoring of the subjective level of daily physical activities over the last seven days (total time spent in light, moderate and strenuous activities) (see also supplementary file 1). This PROM has been developed for this study and is assessed at T_0_, T_1_, T_2_ and T_3_.

##### Behaviour change in energy management

Behaviour changes in patient’s management of their available energy are assessed with the Behaviour Change in Energy Management-Survey (BCEM-S). This survey has been developed for this study based on the energy conservation strategy survey [42, 43] (see also supplementary file 2) and is sent only to the participants from the EG. The survey evaluates which of the 13 energy conservation strategies have become part of the habits and routines of the IEME participants. The aim is to determine which factors prevent implementation of strategies or cause some strategies to be dropped after a brief period. This will provide important information to further improve the IEME program. This survey consists of eight questions and was created with the Qualtrics Research Core software (Qualtrics, Provo, UT, USA) and is sent as a link via e-mail to the participants at T_2_ and T_3_.

### Safety

Tolerability (safety) of the three-week training and education program for PwMS is monitored by continuous measurement of heart rate and a brief scoring of motivation and exhaustion on a 10-point Likert scale after each session (higher values indicate higher exhaustion/motivation). All training sessions are performed as heart-rate-monitored training. Individual thresholds of the participants are determined through CPET (VO_2peak_) and the participants are advised to train in the respective training zone, preventing overtraining of the participants. Heart rate (beats per minute; bpm) is monitored continuously and the training sessions are supervised by MS experienced exercise scientists. Heart rate during and after the training sessions in both intervention groups should not overstep the pre-defined threshold (of 220 bpm – age). This allows normalization of symptoms within 30 min after the exercise sessions. Moreover, adherence is recorded for each participant.

Although not expected, any severe adverse events during training will be reported directly to the physician on duty and to the ethics committee. This includes: (i) severe cardiovascular and pulmonary diseases (renal failure, hepatic dysfunction, cardiovascular disease) and (ii) severe cardiovascular exacerbations (e.g. arterial blood pressure after Riva Rocci > 240/120, heart rate above the age-predicted maximum of 220 – age).

Regarding the education interventions (IEME or PMR) no specific adverse events are expected. If adverse events occur they will be reported directly to the PI and to the ethics committee.

All participants are inpatients at the Valens clinic, Switzerland for the intervention period. Insurance to cover for harms associated with the study protocol is provided for all participants by the Valens clinic, Switzerland, via the Basler Versicherungsgesellschaft, Aeschengraben 21, 4002 Basel.

### Data management and confidentiality

All data collected from paper forms are archived on a personalised desktop-computer with access to the internal IT-network of the Valens clinic. Data is password secured and with the attachment to the Valens network backups are performed every hour. The original study forms are kept in a closet only accessible to the study staff at the Valens clinic. After study termination, paper forms and electronic files are archived for a minimum of 10 years and then destroyed or deleted. The blood samples are disposed after analysis.

Data generation, transmission, storage and analysis of all health-related personal data and the storage of the biological samples within this project will strictly follow the current Swiss legal requirements for data protection. All health-related personal data captured during this project and the biological samples from participants are strictly confidential and disclosure to third parties is prohibited; coding will safeguard participants’ confidentiality.

### Sample size calculation

The a priori sample size calculation was conducted using G*Power 3.1.9.2 software [44]. It was carried out for the investigation of a between-group difference in the primary endpoint (changes in the HRQoL (PCS and MCS) from baseline to T_3_) in an analysis of covariance model (ANCOVA).

Based on our IEME feasibility randomized clinical trial [16] with four months follow-up of HRQoL, we assume a medium between-group effect size of d = 0.5 on changes in the primary endpoint HRQoL. Level of significance (α) with Bonferroni for multiple comparisons (two primary outcomes) was set at 0.025 and power (1–β) at 0.8. According to Borm et al. 2007 [45], the required sample size for the ANCOVA model was calculated with the formula (1–ρ^2^)*n. A correlation between participant’s scores at baseline and at T_3_ of ρ = 0.65 was used. The calculation revealed that 90 participants (EG: 45 / CG: 45) would need to be recruited. Based on previous studies that apply similar interventions [16, 22], we have allowed for 18% dropout and plan to recruit 106 participants.

### Statistical analysis

All analyses are conducted according to intention-to-treat. To determine potential effects of between-subjects factor intervention (IEME + HIIT vs. PMR + MCT) and within-subjects factor measurement time-point (T_0_ to T_3_) and their interaction on primary and secondary outcomes, 2 × 4 mixed ANCOVAs will be applied. As covariates, participants’ baseline score of each specific parameter, EDSS score, MS-phenotype, education, smoking status, occupation, time since diagnosis, age and sex will be included into the model. Post hoc Bonferroni corrected pairwise comparisons will be conducted in case of significant main effect of within-subjects factor measurement time-point. Simple effects analyses will be performed to investigate if groups differ at each measurement time-point. As effect size measures, Eta-square and Cohen’s d will be used in ANCOVA and post hoc analyses, respectively. In addition, bivariate correlation analyses will be conducted to determine potential associations between changes in HRQoL (PCS and MCS) and biomarkers/cardiorespiratory fitness/patient-reported outcomes from T_0_ to T_3_.

Statistical analyses will be performed by a statistician blinded to treatment groups, using SPSS 25® (IBM®, Armonk, NY, USA). A result will be considered significant at *p*-value ≤0.05. Because of multiple testing of the primary endpoint, alpha is adjusted in this case and a *p*-value ≤0.025 will be considered significant.

## Discussion

This study provides new information on a multimodal therapy approach that combines training and education. For many PwMS, fatigue is the most disabling symptom with a strong impact on HRQoL [5, 46]. Many patients who return home from inpatient rehabilitation (subjectively) report that, after rehabilitation, they are not able to integrate and maintain regular training sessions as part of their daily routine. Being too tired, lack of time and impairment are the most reported barriers for implementing training sessions on a regular basis among PwMS [47, 48]. The energy management program will help the participants to better balance, plan and organize regular sequences of moderate physical activity and exercise in their daily life. It may sound controversial to combine a fatigue management program with high-intensive training that leads to transient exhaustion, but the balance between training and recovery is crucial to build up long-term fitness and thus to have more energy available for an active and satisfying everyday life.

Combining these two therapy approaches may help PwMS to maintain greater physical activity levels after inpatient rehabilitation during everyday life, leading to a sustained increase in HRQoL. Secondary objectives are measured to provide further information on the potential benefits from the endurance exercise modalities and educational interventions. Moreover, they will be captured to identify potential correlations with changes in participants’ HRQoL and to increase knowledge of the impact of a multimodal therapy approach on various aspects relevant to participation. Here, the additional information from after participants return home (T_2_, T_3_) will shed light on the reasons why they are able to apply the energy conservation behaviours and on any barriers or facilitators experienced after they return home.

Recent studies have shown that, in contrast to MCT, HIIT significantly improved aerobic capacity (VO_2peak_), serum levels of MMP-2 and verbal memory [22]. Our feasibility study comparing IEME with PMR has shown a significantly higher level of perceived physical functioning and a significant effect on self-efficacy in performing energy conservation strategies due to the education intervention [16]. In both studies inclusion rates and compliance with the study protocol were high and dropout rates were negligible. HIIT and IEME show effects in different domains of HRQoL. This multidisciplinary study will increase our understanding of detailed exercise training recommendations for PwMS during inpatient rehabilitation and beyond.

## Supplementary Information

**Additional file 1.**

**Additional file 2.**

## Data Availability

Not applicable.

## Abbreviations

ANCOVA: Analysis of covariance
BCEM-S: Behavior Change in Energy Management-Survey
CG: Control group
CPET: Cardiopulmonary exercise test
EDSS: Expanded Disability Status Scale
EG: Experimental group
ELISA: Enzyme-linked immunosorbent assay
FSMC: Fatigue Scale of Motor and Cognitive function
HADS: Hospital Anxiety and Depression Scale
HIIT: High-intensity interval training
HR_peak_: Peak heart rate
HRQoL: Health-related quality of life
IEME: Inpatient energy management education
IFN-γ: Interferon-gamma
IL-6: Interleukin-6
KA: Kynurenic acid
KYN: Kynurenine
MCS: Mental Component Summary
MMP-2: Matrix metalloproteinase-2
MMSE: Mini-Mental State Examination
MS: Multiple sclerosis
NLR: Neutrophil-to-lymphocyte ratio
OSA: Occupational self-assessment
PCS: Physical Component Summary
PI: Principal investigator
PMR: Progressive muscle relaxation
PROM: Patient-reported outcome measure
PwMS: Persons with multiple sclerosis
rpm: Revolutions per minute
QA: Quinolinic acid
SEPECSA: Self-efficacy of perceived energy conservation strategies assessment
SF-36: Medical Outcome Study 36-item Short Form Health Survey
TRP: Tryptophan
VO_2peak_: Peak oxygen consumption
Watt_peak_: Peak wattage
