# Supplementary material for: High-intensity interval training and energy management education, compared with moderate continuous training and progressive muscle relaxation, for improving health-related quality of life in persons with multiple sclerosis: study protocol of a randomized controlled superiority trial with six months’ follow-up
Source: BMC Neurol. 2021 Feb 11;21:65. doi: 10.1186/s12883-021-02084-0 (PMC7877079; doi:10.1186/s12883-021-02084-0)
Supplement: Supplementary file 1 — Additional file 1. [file 12883_2021_2084_MOESM1_ESM.docx]

**Physical activity level questionnaire**

With this questionnaire we would like to know how active you have been **in the last 7 days**. The questions refer to the physical activity level.

| 1. This question is about your overall activity level:  **In relation to the last 7 days**: how would you rate your **overall activity level**, i.e. everyday activity + physical activity + sporting activity + occupational exertion?   - Very low - Rather low - Mediocre - Rather high - Very high |
| --- |

| 2. With this question we would like to find out **how long** you do the following **physical everyday activities/homework (excluding sports and therapy)** on average per day.  **Referring to the last 7 days**: **how many minutes per day on average** did you do the following activities (excluding sports and therapy)?   - **Light physical activity** (e.g. doing laundry, cooking, washing dishes, taking a shower, walking a short distance/moving around the house) - Less than 15min per day - 15 – 30min per day - 30 – 45min per day - 45 – 60min per day - More than 60min per day - **Moderately strenuous physical activity** (e.g. doing small shopping, playing with children, meeting friends, longer walk e.g. with the dog, mowing the lawn, moderate gardening) - Less than 15min per day - 15 – 30min per day - 30 – 45min per day - 45 – 60min per day - More than 60min per day - **Very strenuous physical activity** (e.g. very heavy gardening, cleaning windows, doing big shopping, vacuuming, going to therapy, washing the car, carrying wood, making fresh beds) - Less than 15min per day - 15 – 30min per day - 30 – 45min per day - 45 – 60min per day - More than 60min per day |
| --- |

| 3. With this question we would like to know **how long** you do the following **sporting activities** on average per day.  **In relation to the last 7 days: how many minutes per day on average** did you do the following sporting activities?   - **Light activity (low exertion)** (e.g. golf, walking, loose cycling, yoga) - Less than 15min per day - 15 – 30min per day - 30 – 45min per day - 45 – 60min per day - More than 60min per day - **Moderately strenuous activity (not exhausting)** (e.g. walking, moderate cycling, brisk walking, dancing) - Less than 15min per day - 15 – 30min per day - 30 – 45min per day - 45 – 60min per day - More than 60min per day - **Very strenuous activity (heart beating fast)** (e.g. weight training, fast walking, strenuous cycling, strenuous swimming, aerobics) - Less than 15min per day - 15 – 30min per day - 30 – 45min per day - 45 – 60min per day - More than 60min per day |
| --- |

Thank you!
