# Supplementary material for: High-intensity interval training and energy management education, compared with moderate continuous training and progressive muscle relaxation, for improving health-related quality of life in persons with multiple sclerosis: study protocol of a randomized controlled superiority trial with six months’ follow-up
Source: BMC Neurol. 2021 Feb 11;21:65. doi: 10.1186/s12883-021-02084-0 (PMC7877079; doi:10.1186/s12883-021-02084-0)
Supplement: Supplementary file 2 — Additional file 2. [file 12883_2021_2084_MOESM2_ESM.docx]

**Behaviour change in energy management**

**Online survey for IEME-participants**

With this questionnaire we would like to find out whether and, if so, how your everyday life has changed afterwards. It is about how you deal with your energy after participating in the energy management education.

1. Which of the statements below best applies to you at the moment?

*Please tick only one statement.*

- I occasionally think about changing daily routines and habits or using energy conservation strategies.
- I have tried to use **some** energy conservation strategies.
- I have started to integrate **selected** energy conservation strategies into my daily routines and habits as a permanent feature.
- I have been using **certain** energy conservation strategies regularly and stably since I completed the energy management education.
- I have given up trying to change daily routines and habits or to use energy conservation strategies.

1. Which of the following strategies **were you already using consciously before** attending the energy management education program?

***Several*** *answers are possible.*

- I did not consciously use any strategies before participating in the energy management education
- Adjust the height of the workstation or the place where I carry out an activity
- Place utensils, appliances and furniture differently
- Use aids and assistive devices
- Adopt ergonomic postures
- Omit partial steps of activities
- Delegate activities
- Simplifying/modifying the execution of activities
- Communicate own needs to others
- Consciously set priorities
- Consciously balance work, compulsory tasks and free time
- Shifting the timing of activities
- Consciously plan breaks in the daily routine or weekly schedule
- Take breaks before or during strenuous activities
- Other strategies……………….

1. Which of the following strategies have you tried in addition since returning home?

*(Only the strategies that were NOT indicated in question 2 appear.)*

***Several*** *answers are possible.*

- I have not tried any additional strategies
- Adjust the height of the workstation or the place where I carry out an activity
- Place utensils, appliances and furniture differently
- Use aids and assistive devices
- Adopt ergonomic postures
- Omit partial steps of activities
- Delegate activities
- Simplifying/modifying the execution of activities
- Communicate own needs to others
- Consciously set priorities
- Consciously balance work, compulsory tasks and free time
- Shifting the timing of activities
- Consciously plan breaks in the daily routine or weekly schedule
- Take breaks before or during strenuous activities
- Other strategies……………….

1. Which of the following strategies do you **currently/now regularly** use in your everyday life?

***Several*** *answers are possible.*

- I do not use any strategy regularly.
- Adjust the height of the workstation or the place where I carry out an activity
- Place utensils, appliances and furniture differently
- Use aids and assistive devices
- Adopt ergonomic postures
- Omit partial steps of activities
- Delegate activities
- Simplifying/modifying the execution of activities
- Communicate own needs to others
- Consciously set priorities
- Consciously balance work, compulsory tasks and free time
- Shifting the timing of activities
- Consciously plan breaks in the daily routine or weekly schedule
- Take breaks before or during strenuous activities
- Other strategies……………….

| 1. **From your experience, how helpful are the energy conservation strategies you use in your everyday life?**   Please answer the question only in relation to the strategies you use.  Please cross out the energy saving strategies that you **do not use**.  Please tick the number that corresponds to your experience (1) not at all helpful <-----------> very helpful (10) | | | | | | | | | | | |
| --- | --- | --- | --- | --- | --- | --- | --- | --- | --- | --- | --- |
| Adjust the height of the workstation or the place where I carry out an activity | 1 | 2 | 3 | 4 | 5 | 6 | 7 | 8 | 9 | 10 |  |
| Place utensils, appliances and furniture differently | 1 | 2 | 3 | 4 | 5 | 6 | 7 | 8 | 9 | 10 |  |
| Use aids and assistive devices | 1 | 2 | 3 | 4 | 5 | 6 | 7 | 8 | 9 | 10 |  |
| Adopt ergonomic postures | 1 | 2 | 3 | 4 | 5 | 6 | 7 | 8 | 9 | 10 |  |
| Omit partial steps of activities | 1 | 2 | 3 | 4 | 5 | 6 | 7 | 8 | 9 | 10 |  |
| Delegate activities | 1 | 2 | 3 | 4 | 5 | 6 | 7 | 8 | 9 | 10 |  |
| Simplifying/modifying the execution of activities | 1 | 2 | 3 | 4 | 5 | 6 | 7 | 8 | 9 | 10 |  |
| Communicate own needs to others | 1 | 2 | 3 | 4 | 5 | 6 | 7 | 8 | 9 | 10 |  |
| Consciously set priorities | 1 | 2 | 3 | 4 | 5 | 6 | 7 | 8 | 9 | 10 |  |
| Consciously balance work, compulsory tasks and free time | 1 | 2 | 3 | 4 | 5 | 6 | 7 | 8 | 9 | 10 |  |
| Shifting the timing of activities | 1 | 2 | 3 | 4 | 5 | 6 | 7 | 8 | 9 | 10 |  |
| Consciously plan breaks in the daily routine or weekly schedule | 1 | 2 | 3 | 4 | 5 | 6 | 7 | 8 | 9 | 10 |  |
| Take breaks before or during strenuous activities | 1 | 2 | 3 | 4 | 5 | 6 | 7 | 8 | 9 | 10 |  |

| 1. **How easy was it to integrate these energy conservation strategies into your everyday life?**   Please answer the question only in relation to the strategies you use.  Please cross out the energy saving strategies that you **do not use**.  Please tick the number that corresponds to your experience (1) not at all easy <--------------------> very easy (10) | | | | | | | | | | | |
| --- | --- | --- | --- | --- | --- | --- | --- | --- | --- | --- | --- |
| Adjust the height of the workstation or the place where I carry out an activity | 1 | 2 | 3 | 4 | 5 | 6 | 7 | 8 | 9 | 10 |  |
| Place utensils, appliances and furniture differently | 1 | 2 | 3 | 4 | 5 | 6 | 7 | 8 | 9 | 10 |  |
| Use aids and assistive devices | 1 | 2 | 3 | 4 | 5 | 6 | 7 | 8 | 9 | 10 |  |
| Adopt ergonomic postures | 1 | 2 | 3 | 4 | 5 | 6 | 7 | 8 | 9 | 10 |  |
| Omit partial steps of activities | 1 | 2 | 3 | 4 | 5 | 6 | 7 | 8 | 9 | 10 |  |
| Delegate activities | 1 | 2 | 3 | 4 | 5 | 6 | 7 | 8 | 9 | 10 |  |
| Simplifying/modifying the execution of activities | 1 | 2 | 3 | 4 | 5 | 6 | 7 | 8 | 9 | 10 |  |
| Communicate own needs to others | 1 | 2 | 3 | 4 | 5 | 6 | 7 | 8 | 9 | 10 |  |
| Consciously set priorities | 1 | 2 | 3 | 4 | 5 | 6 | 7 | 8 | 9 | 10 |  |
| Consciously balance work, compulsory tasks and free time | 1 | 2 | 3 | 4 | 5 | 6 | 7 | 8 | 9 | 10 |  |
| Shifting the timing of activities | 1 | 2 | 3 | 4 | 5 | 6 | 7 | 8 | 9 | 10 |  |
| Consciously plan breaks in the daily routine or weekly schedule | 1 | 2 | 3 | 4 | 5 | 6 | 7 | 8 | 9 | 10 |  |
| Take breaks before or during strenuous activities | 1 | 2 | 3 | 4 | 5 | 6 | 7 | 8 | 9 | 10 |  |

| 1. What are the reasons why you have not integrated this strategy into your daily life?   *Please tick the answer that best applies to you.*  *(Only the strategies that were NOT indicated in question 4 appear.)* | | Not convinced it would make a difference | Forgotten to try out | Lack of practical help from third parties | Unsure how to realise / make, how to implement | Postponed until later | Lack of moral support in the personal / professional environment | Is practically not feasible | Lack of financial resources | Does not fit into my complex everyday life | Do not have access to the equipment / assistive devices | Andere Gründe |
| --- | --- | --- | --- | --- | --- | --- | --- | --- | --- | --- | --- | --- |
| 1 | Adjust the height of the workstation or the place where I carry out an activity |  |  |  |  |  |  |  |  |  |  |  |
| 2 | Place utensils, appliances and furniture differently |  |  |  |  |  |  |  |  |  |  |  |
| 3 | Use aids and assistive devices |  |  |  |  |  |  |  |  |  |  |  |
| 4 | Adopt ergonomic postures |  |  |  |  |  |  |  |  |  |  |  |
| 5 | Omit partial steps of activities |  |  |  |  |  |  |  |  |  |  |  |
| 6 | Delegate activities |  |  |  |  |  |  |  |  |  |  |  |
| 7 | Simplifying/modifying the execution of activities |  |  |  |  |  |  |  |  |  |  |  |
| 8 | Communicate own needs to others |  |  |  |  |  |  |  |  |  |  |  |
| 9 | Consciously set priorities |  |  |  |  |  |  |  |  |  |  |  |
| 10 | Consciously balance work, compulsory tasks and free time |  |  |  |  |  |  |  |  |  |  |  |
| 11 | Shifting the timing of activities |  |  |  |  |  |  |  |  |  |  |  |
| 12 | Consciously plan breaks in the daily routine or weekly schedule |  |  |  |  |  |  |  |  |  |  |  |
| 13 | Take breaks before or during strenuous activities |  |  |  |  |  |  |  |  |  |  |  |

| 1. Lastly, we would like to know how much **influence** you think certain conditions have on your everyday life.   *Please tick the answer that corresponds to you.*  **It has** | **no influence,** | **a little influence,** | **a mediocre influence,** | **quite an influence,** | **a strong influence,** |
| --- | --- | --- | --- | --- | --- |
| 1. **when** people in my personal environment understand the importance of energy saving strategies. |  |  |  |  |  |
| 1. **when** people in my professional environment know how to support me in dealing with my energy. |  |  |  |  |  |
| 1. **when** people around me know what consequences fatigue has on my quality of life. |  |  |  |  |  |
| 1. **when** people around me know how fatigue affects my performance. |  |  |  |  |  |
| 1. **when** I have contact with people with similar experiences. |  |  |  |  |  |
| 1. **when** my doctor knows what my goals are in everyday life. |  |  |  |  |  |
| 1. **when** my family knows what to do and how to support me. |  |  |  |  |  |
| 1. **wenn** meine Familie weiss, welche Strategien ich versuche anzuwenden. |  |  |  |  |  |
| 1. **when** I have easy access to professional support (e.g. occupational therapy, physiotherapy, psychotherapist, social worker, …). |  |  |  |  |  |
| 1. **when** I have easy access to support services (Spitex, driving service, …). |  |  |  |  |  |
| 1. **when** I have access to information about assistive devices, technologies and useful equipment. |  |  |  |  |  |
| 1. **when** my environment if informed about fatigue. |  |  |  |  |  |

**Thank you!**
